# Slow–fast control of eye movements: an instance of Zeeman’s model for an action

**DOI:** 10.1007/s00422-020-00845-7

**Published:** 2020-09-30

**Authors:** Richard A. Clement, Ozgur E. Akman

**Affiliations:** College of Engineering, Computing and Mathematics, University of Exeter, North Park Road, Exeter, EX4 4QF UK

**Keywords:** Eye movements, Saccades, Main sequence, Omnipause neurons, Slow–fast systems, Nonlinear dynamics

## Abstract

The rapid eye movements (saccades) used to transfer gaze between targets are examples of an action. The behaviour of saccades matches that of the slow–fast model of actions originally proposed by Zeeman. Here, we extend Zeeman’s model by incorporating an accumulator that represents the increase in certainty of the presence of a target, together with an integrator that converts a velocity command to a position command. The saccadic behaviour of several foveate species, including human, rhesus monkey and mouse, is replicated by the augmented model. Predictions of the linear stability of the saccadic system close to equilibrium are made, and it is shown that these could be tested by applying state-space reconstruction techniques to neurophysiological recordings. Moreover, each model equation describes behaviour that can be matched to specific classes of neurons found throughout the oculomotor system, and the implication of the model is that build-up, burst and omnipause neurons are found throughout the oculomotor pathway because they constitute the simplest circuit that can produce the motor commands required to specify the trajectories of motor actions.

## Introduction

The rapid eye movements that transfer gaze from one object to another are referred to as saccades. These discrete behaviours show a relatively invariant relationship between the size of the movement and its peak velocity and duration. The peak velocity of human saccades typically varies from 30 to 700 $^{\circ }$ s$^{-1}$ and their duration varies from 30 to 100 ms for eye movements of 0.5$^{\circ }$–40$^{\circ }$ in amplitude. The peak velocity progressively saturates with increasing saccade amplitude after 20$^{\circ }$ and the duration increases linearly with amplitude. These consistent relationships between the amplitudes, durations and peak velocities are referred to as the main sequence  (Bahill et al. 1975).

The most direct neural pathway for the control of saccades runs from the retina to the superior colliculus, then on to the brainstem, and terminates at the oculomotor nuclei. At the physiological level, neuronal recordings in awake animals have provided detailed information about the circuitry involved in the generation of saccades. The motoneurons that innervate the extraocular muscles are located in the nuclei of cranial nerves III, IV and VI and have a burst-tonic pattern of discharge. The frequency of the burst of activity is correlated with eye velocity during the saccade, and the level of tonic activity is correlated with the eye position at the end of the saccade. The two components are coordinated by neural integration of a velocity command produced by burst neurons in the brainstem. Burst neurons fire during saccadic movements in a preferred direction (their on direction) and are silent for movements in the opposite direction (their off direction). They can be classified into long-lead burst neurons, which steadily increase their firing before a saccade, and medium-lead burst neurons, which only begin firing shortly before the start of the saccade. In addition to the burst units in the brainstem, there is also a class of cells referred to as omnipause neurons. These fire continuously except just before and during saccades in any direction, during which time they cease firing (Scudder et al. 2002; Sparks 2002). A striking feature of these cells is that if they are electrically stimulated during a saccade, then the saccade halts while the stimulation is applied, but continues unaffected as soon as the stimulation ceases (Keller 1974).

A key insight into the mechanistic behaviour of the saccadic system was that signals from the visual pathway arrive after the majority of saccades have already terminated and so cannot be used for saccadic control. This led to the proposal that a local feedback signal from the burst generator is used to control the saccade. Initially, this local feedback signal was assumed to be the position of the eye with respect to the head taken from the output of the neural integrator, and subsequently, it was assumed to be the displacement of the eye during the saccade computed by integrating the output of the burst neurons. The essential control loop consists of a required eye displacement signal that is fed into the burst neurons, which in turn produce an eye velocity command. The command is a saturating, nonlinear function of the motor error, computed as the difference between the current eye position and the target eye position. The velocity command is integrated to obtain an estimate of the current eye displacement, and this is subtracted from the required eye displacement to obtain an estimate of the motor error. More recently, it has been found that the superior colliculus provides signals that specify the kinematics of the eyes throughout a saccade and not just the endpoints of the movement (Goossens and Van Opstal 2006; Smalianchuk et al. 2018).

The dynamics associated with discrete behavioural events, or *actions*, of which saccades are an example, have three distinctive features. Firstly, there is a starting position that corresponds to a stable equilibrium point of the system. Secondly, there is a switching mechanism that initiates the movement by shifting the state of the system away from the equilibrium. Thirdly, there is a trajectory that returns the system to the equilibrium. Zeeman (1977) formulated a generic system of three differential equations that capture the required features of the trajectory.

In this paper, we test whether this generic system of equations is applicable to saccadic eye movements, beginning with the hypothesis that the positive values of the variables *x*, *y* and *z* in Zeeman’s model correspond to the firing rates of the long-lead, medium-lead and omnipause cells in the brainstem, respectively. We then augment Zeeman’s equations to model the charging process associated with the first stage of the saccade cycle, during which the specification of the required eye displacement builds up before the movement can start (Gancarz and Grossberg 1998). Next, we introduce an additional equation representing the neural integrator, enabling us to compare model simulations with experimental eye movement data. We show that the resulting model successfully accounts for the saccadic behaviour of several species for which quantitative data on the oculomotor system are available and that it is also compatible with the distinctive properties of the omnipause cells. We conclude by exploring the testable neurophysiological implications of the model.

## Methods and results

### Zeeman’s equations

**Fig. 1:**
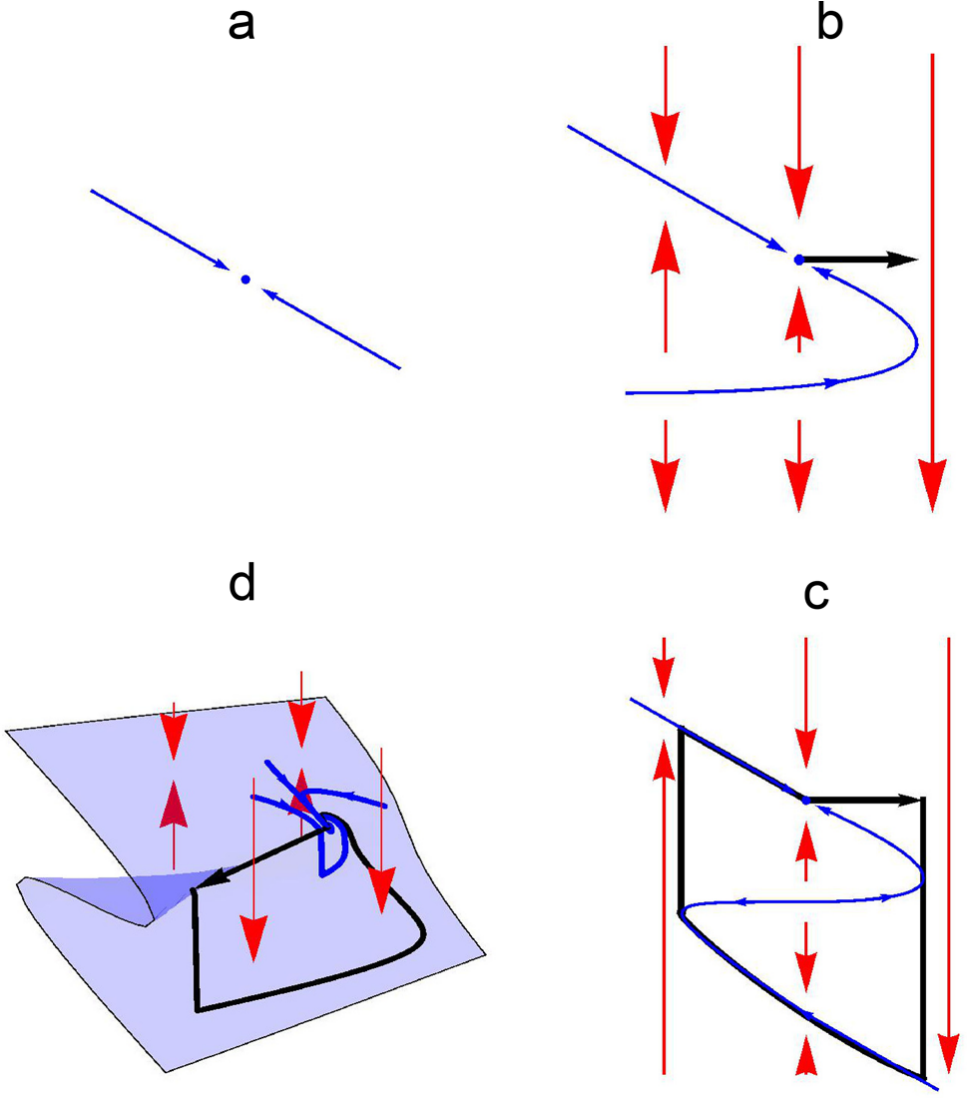
Geometric derivation of the slow–fast description of an action. **a** State-space diagram of a one-dimensional system with a stable equilibrium. **b** Addition of a transverse vector field generates threshold behaviour when the state of the one-dimensional system moves beyond the fold. **c** Incorporation of an additional fold results in a return to equilibrium. **d** A smooth return can be ensured by using a folded surface (the slow manifold)

Here, we briefly review Zeeman’s geometric argument for the derivation of his model of an action. This is based on the dynamical systems framework, in which the state of the system is represented by a point in a state space that moves along a trajectory determined by the vector field defined on the space. Zeeman modelled the action’s stable equilibrium by a one-dimensional system with a stable fixed point, as shown in Fig. 1a. Secondly, to incorporate the trigger property, he made a fold in the line representing the one-dimensional system and set up a two-dimensional vector field transverse to the line so that if the state of the system is displaced beyond the fold, then the vector field carries the state away from the stable equilibrium. This mechanism is illustrated in Fig. 1b. Finally, the return to the stable equilibrium was incorporated by folding the line of the one-dimensional system into an S-shape so that the state is guided back to the equilibrium point. The complete cycle is illustrated in Fig. 1c.

Zeeman further noted that in the two-dimensional system shown in Fig. 1c, the return to equilibrium requires a jump to mirror the jump associated with the trigger. In three dimensions, however, a folded surface can be used to obtain a smooth return to equilibrium, as illustrated in Fig. 1d. Zeeman’s generic equations for describing such an action (Zeeman 1977) are

1 $$\begin{aligned} \begin{aligned} \lambda \dot{x}&= -y-1, \\ \lambda \dot{y}&= -y-z, \\ \lambda \epsilon \dot{z}&= -(z^{3}+yz+x). \end{aligned} \end{aligned}$$

Although this system of equations has only one nonlinearity, it has the required properties for generating an action with a smooth return. The unit constant in the equation for the rate of change of the *x* variable ensures the system has a stable equilibrium point at $(0,-\,1,1)$ that is a spiral attractor and a transverse vector field is generated by the equation for the *z* variable. The behaviour of the *z* variable evolves on a much faster timescale than that of the *x* and *y* variables, so *z* is referred to as a fast variable and *x* and *y* are referred to as slow variables. The parameter $\epsilon$ determines the timescale on which the fast variable evolves. If one assumes that the magnitude of the derivative of the fast variable is of order 1, then with a small value of $\epsilon$, the equation for the fast variable can be approximated by the cubic equation

2 $$\begin{aligned} 0 = -(z^{3}+yz+x). \end{aligned}$$

**Fig. 2:**
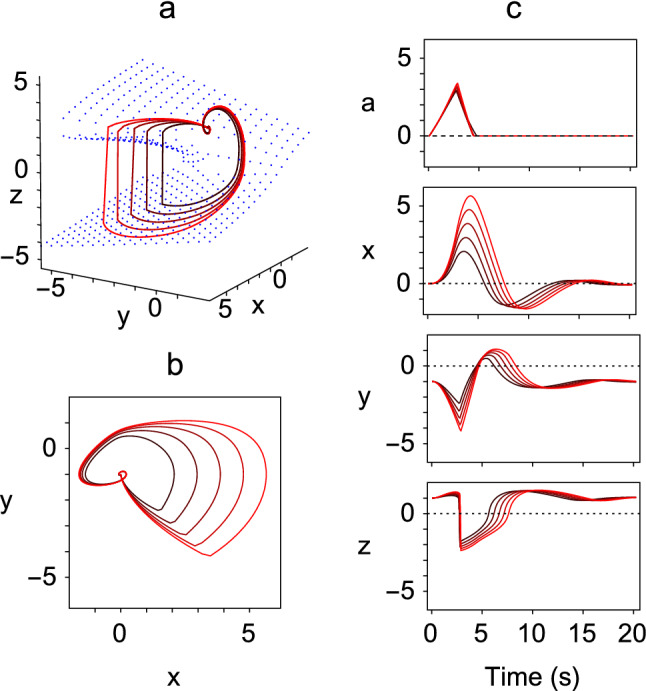
Example solutions of the augmented Zeeman system of Eq. (3) generated with $\lambda =1$. Each of the trajectory loops starts from and finishes at the equilibrium state $(0,0,-1,1)$ and corresponds to a single action. The lengths of the trajectories depend on the values of the $\mu$ parameter, and in this figure the 5 trajectories were generated by the values $\mu =0.721, 0.930,1.089,1.224, 1.343$. (Action length increases with $\mu$.) In this, and all other figures, $\epsilon =0.01$. **a** Phase space plot showing trajectories of the *x*, *y* and *z* variables, in which the slow manifold is indicated by blue dots. **b** Projections of trajectories onto the (*x*, *y*) plane. **c** Time series plots of *a*, *x*, *y* and *z*, showing the characteristic behaviour of each variable throughout the range of values of $\mu$ (colour figure online)

This equation describes a folded surface in (*x*, *y*, *z*) space, referred to as the slow manifold. An action is deemed to occur when the state of the system moves over the fold and drops rapidly back onto the slow manifold (Figs. 1d, 2a). This rapid change in state models the switching that occurs at the onset of an action. Note that the time constant $\lambda$ in Eq. (1) was not part of Zeeman’s original description of the equations but has been added here in order to be able to fit the time course of the action to those of experimentally recorded eye movements (Zeeman’s original system corresponds to the case $\lambda =1$).

Zeeman used his two-dimensional model of an action to describe the generation of the heartbeat and his three-dimensional model of an action to simulate the nerve impulse (Zeeman 1977). Although the description of the heartbeat proved useful for generating synthetic heartbeat waveforms, that of the nerve impulse was not in agreement with experiments. This was due to the fact that the latter used a value of 0.8 for the parameter specifying the ratio of the slow to the fast behaviours, and for the distinction between slow and fast behaviours to be valid, this parameter must be much smaller than 1 (Stewart and Woodcock 2006).

Another application of the two-dimensional model was made later by Jirsa and Kelso (2005) who reprised the geometric argument in order to demonstrate that the two-dimensional system could underly both oscillatory and discrete behaviour. The behaviour of both the two- and three-dimensional systems has subsequently been analysed in detail. It has been found that in the two-dimensional system, the transition between discrete and oscillatory behaviour is controlled by the fast variable parameter $\epsilon$ (Slowiński et al. 2018), whilst in the three-dimensional system, the form of the action changes for small actions characterised by trajectories that stay close to the fold line and equilibrium position (Broer et al. 2013). It is therefore of interest to have another application of the geometric description of an action where the predictions of the equations can be explored.

### Extending Zeeman’s equations to include an accumulator unit

As it stands, Zeeman’s model of an action is incomplete because it does not include the mechanism involved in making the decision to execute an action. Such decisions involve a trade-off between making a rapid but inaccurate action and a slow but accurate one. A mechanism that has been shown to solve this problem in a wide variety of biological situations consists of an accumulator unit that integrates evidence for an action until a threshold is reached, at which point the action is initiated (Marshall et al. 2009). The addition of such a unit leads to the augmented system of four equations below:

3 $$\begin{aligned} \begin{aligned} \lambda \dot{a}&= H(a)z, \\ \lambda \dot{x}&= -y-1, \\ \lambda \dot{y}&= -y-z -\mu a,\\ \lambda \epsilon \dot{z}&= -(z^{3}+y z+x). \end{aligned} \end{aligned}$$

Here, *a* is the accumulator variable, *H*(*a*) denotes the Heaviside function ($H(a)=0$ for $a\le 0$ and $H(a)=1$ for $a>0$), and $\mu$ is a positive constant that determines how far along the fold the system is when the action is initiated. Multiplying *H*(*a*) by the *z* variable is a simple method for ensuring that every time an action occurs, the accumulator is reset to zero because in the resting state the value of *z* is unity and so has no effect on the accumulator build-up, but once the action begins it turns negative, driving *a* back towards 0. Without this modification, the equations generate cyclical behaviour because after every action is completed, the accumulator output again rises steadily, forcing the system to the threshold for an action.

The behaviour of the *x*, *y* and *z* variables for various values of the parameter $\mu$, corresponding to actions of different amplitudes, are plotted as phase space trajectories in Fig. 2a. Also shown in Fig. 2b is the projection of the trajectories onto the (*x*, *y*) plane. In Fig. 2c, the timeseries of the variables are plotted. These show build-up (*x*), bursting (*y*) and pausing (*z*) patterns of firing during the action that are characteristic of the neurons found throughout the saccadic eye movement pathways (Moschovakis et al. 1996).

### Slow–fast models of saccadic control

**Fig. 3:**
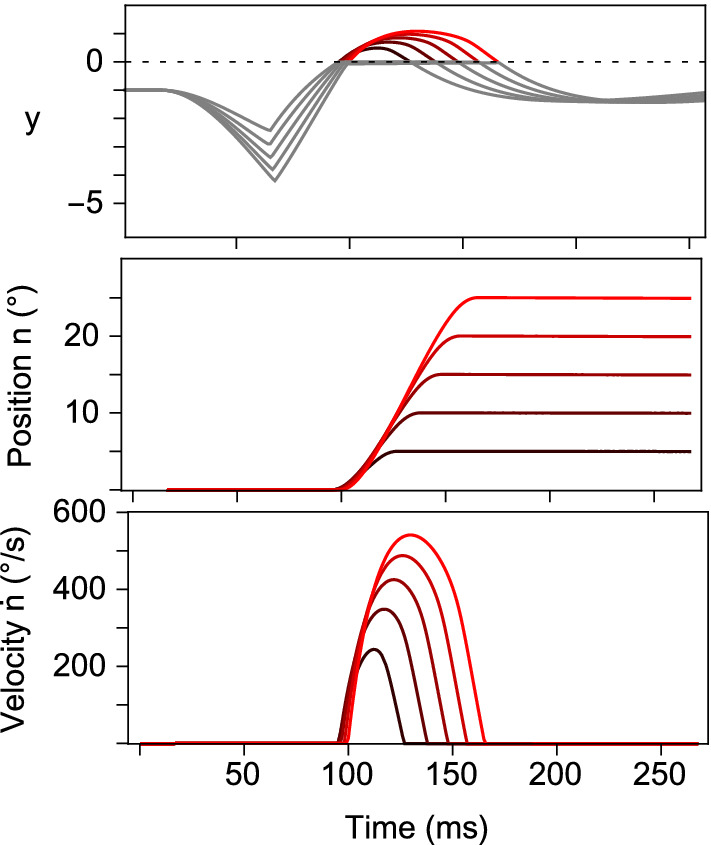
Example saccades generated by Model 1, specified by the system of Eq. (4). The values of $\mu$ were the same as those used in Fig. 2. (Saccade amplitude increases with $\mu$.) The parameters $\lambda = 0.018$ and $\kappa = 500$ were selected to produce saccades that matched those of humans. The position of the eye is given by the output of the oculomotor neural integrator *n* and the velocity of the eye is given by its derivative $\dot{n}$. The simulated eye velocity is very similar, but not identical, to the velocity command $\kappa y^{+}$, because the neural integrator is leaky

To convert the augmented equations (3) into a model of the brainstem saccade controller, we began by assuming that the instantaneous level of burst cell firing, specified by the positive values $y^{+}$ of the *y* variable, is linearly related to the velocity of the eye, as has been found experimentally (Cullen and Guitton 1997). Accordingly, the oculomotor neural integrator signal *n* was modelled as a leaky integrator of the velocity command $y^{+}$, with a long time constant $T_n$, reflecting the slow drift of the eyes back to the straight-ahead position in the dark (Becker and Klein 1973). It follows that as the value of the *n* variable corresponds to the position of the eye, the system of equations obtained by combining the three slow–fast equations together with an accumulator [Eq. (3)] and a neural integrator

4 $$\begin{aligned} \begin{aligned} \lambda \dot{a}&= H(a)z, \\ \lambda \dot{x}&= -y-1, \\ \lambda \dot{y}&= -y-z -\mu a,\\ \lambda \epsilon \dot{z}&= -(z^{3}+y z+x),\\ \dot{n}&= -\frac{n}{T_{n}}+\kappa y^{+}, \end{aligned} \end{aligned}$$

gives a model of saccadic control, which we refer to hereafter as **Model 1**. The parameter $\kappa$ scales the velocity signal generated by the behavioural model to the eye velocity found experimentally. With the parameter values for the human found in the next section, the five timeseries for the variable *y* illustrated in Fig. 2c generate saccades in the range 5$^{\circ }$–25$^{\circ }$ in 5$^{\circ }$ steps as illustrated in Fig. 3.

Experimental measurements of the orbital muscle plant (a collective term for the eye muscles and orbital tissue) imply that it can be modelled as a linear mechanical system formed of two parallel spring and viscous elements connected in series (Robinson 1964). To ensure that the experimental finding that the saccade velocity profile matches the profile of the neural signal $y^{+}$ holds true, the orbital plant has to be supplied with an appropriate combination of a neural velocity signal, a neural position signal provided by the neural oculomotor integrator and a slide signal to compensate for the slower dynamics of the plant. This signal was assumed to be computed from the velocity command, within the cerebellum (Optican and Miles 1985). Given this assumption, there is no need to include the orbital plant in the model because its dynamics are compensated for subsequent to the output of the model.

To increase the range of possible behaviours generated by the system of slow–fast equations, we altered only the linear behaviour of the Zeeman equations so that the properties required to describe an action remained unaffected. An additional parameter $\theta$ was used to change the eigenvalues at the equilibrium point so that the rate at which trajectories spiral towards the equilibrium could be modified without altering its location. **Model 2** was then defined by the following set of equations:

5 $$\begin{aligned} \begin{aligned} \lambda \dot{a}&= H(a)z, \\ \lambda \dot{x}&= -y-1, \\ \lambda \dot{y}&= -y-z -\mu a,\\ \lambda \epsilon \dot{z}&= -( \theta (z^{3}+y z)+x), \\ \dot{n}&= -\frac{n}{T_{n}}+\kappa y^{+}. \end{aligned} \end{aligned}$$

### Quantitative simulations of main sequence behaviour

Although main sequence plots obtained from voluntary saccades and the quick phases of optokinetic and vestibular nystagmus for an individual are highly correlated, implying a single underlying mechanism, the data show considerable variability between recordings. This is because the peak velocities depend on both the state of alertness of the subject and the task, being highest when an alert subject is looking at a clear target and lowest for a drowsy subject making saccades to a remembered target in the dark (Moschovakis et al. 1996). Nevertheless, comparisons of the eye movements of different species have revealed statistically significant differences (Berg et al. 2009). It is therefore a challenge to see if a single generic model can encompass all the behaviours found in different saccadic systems. We tested data from five species (human, rhesus monkey, cat, rabbit and mouse) for which the time constants of the orbital plant have been determined.

Over a wide range of saccade sizes, both duration and peak velocity are nonlinearly related to saccade amplitude. Here, we have used the amplitude range 5$^{\circ }$–25$^{\circ }$, which avoids the small saccades that are difficult to measure and the large saccades which are beyond the range of animals such as the cat. Within this reduced range, the main sequence data collected in experimental studies have most commonly been described by linear relationships (Fuchs 1967; Takagi et al. 1998—rhesus monkey; Evinger and Fuchs 1978; Blakemore and Donaghy 1980; Guitton et al. 1984; Ruhland et al. 2013—cat; Collewijn 1977—rabbit; Sakatani and Isa 2007; Stahl 2008—mouse). For the purposes of comparison, quantitative descriptions of the main sequence data were formulated and these are given in Table 1. It is clear from the observations made in the preceding paragraph that these curves can only be considered as representative descriptions of the data. Indeed, all the peak velocity curves lie within the range found with a large number of recordings made only from rhesus monkeys (Berg et al. 2009). Our purpose in this section was simply to test how well Model 1 and Model 2 could account for this range of typical behaviours.

**Table 1:** Description of the main sequences obtained from published data for the five species considered in this study

Species	Duration (ms)	Peak velocity ($^\circ$ s$^{-1}$)
Human	$20+2A$	$185+16.6A$
Rhesus	$20+1.3A$	$138+28A$
Cat	$50+3A$	$100+12A$
Rabbit	$52+2A$	$93+9A$
Mouse	$20+0.5A$	$100+50A$

**Fig. 4:**
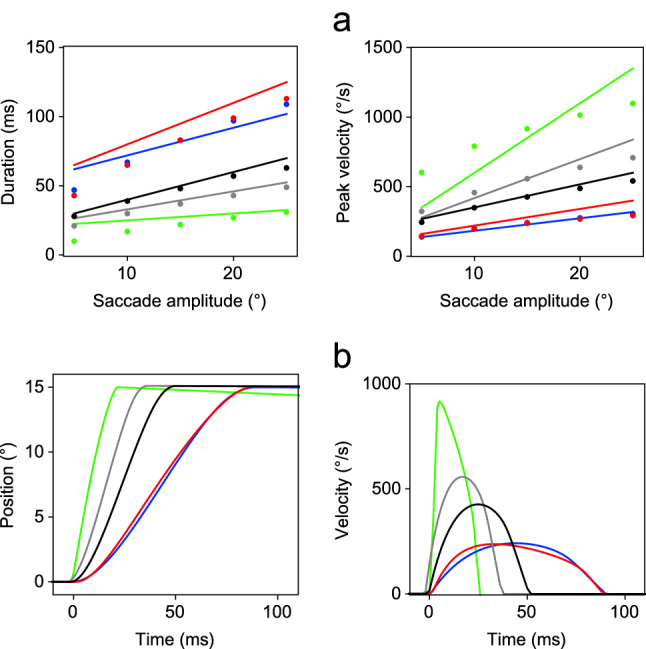
Simulations of the main sequence of saccadic eye movements in five species for Model 1. **a** Plot of the description of the experimental data (continuous lines) together with the simulated data (dots) obtained by using Eq. (4) with the parameter values given in Table 2. The results for different species are indicated by line colour: human (black solid line), rhesus (grey solid line), cat (red solid line), rabbit (blue solid line ) and mouse (green solid line). **b** Simulated eye position (*n*) and velocity ($\dot{n}$) timeseries for a 15$^{\circ }$ saccade in each species (colour figure online)

**Fig. 5:**
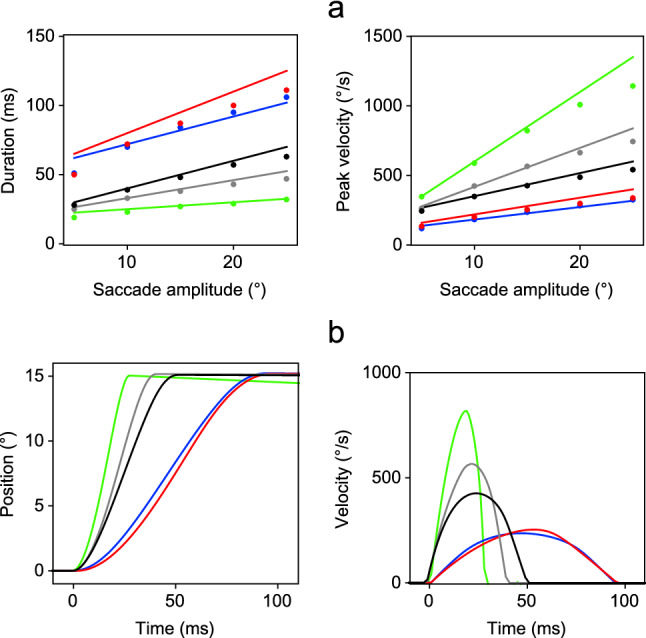
Simulations of the main sequence of saccadic eye movements in five species for Model 2. **a** Plot of the description of the experimental data (continuous lines) together with the simulated data (dots) obtained by using Eq. (5) with the parameter values given in Table 3. The results for different species are indicated by line colour: human (black solid line), rhesus (grey solid line), cat (red solid line), rabbit (blue solid line) and mouse (green solid line). **b** Simulated eye position (*n*) and velocity ($\dot{n}$) timeseries for a 15$^{\circ }$ saccade in each species (colour figure online)

Saccade duration in milliseconds and peak velocity in degrees per second are specified by linear functions of the saccade amplitude *A* in degrees. The graphs of these functions are shown as continuous lines in Figs. 4 and 5

The neural integrator time constant $T_n$ is greater than 20 s for the human (Becker and Klein 1973), rhesus monkey (Cannon and Robinson 1980) and cat (Robinson 1974)—a value of 25 s was therefore used for all these species. As no experimentally determined NI value was available for the rabbit, it was assumed that the time constant was similar to that of the larger mammals. For the mouse, the NI time constant has a smaller value of 2.1 s (van Alphen et al. 2001).

For all simulations shown in this paper, we used a value of 0.01 for the parameter $\epsilon$. We initially ran simulations with an $\epsilon$ value of 0.1, primarily for speed; the results of the simulations were very similar, except for the values of $\mu$ needed to obtain saccades with the correct amplitudes. The values for $\kappa$, $\lambda$ and $\mu$ given in Table 2 were obtained by fitting the equations to the linear description of the main sequence data through grid searching, as follows. Firstly, for each animal, a set of 5 values of $\mu$ were selected that generated a set of saccades spanning the amplitude range 5$^{\circ }$–25$^{\circ }$. The parameters $\lambda$ and $\kappa$ were then varied in steps of 0.001 and 20, respectively, and for each set of parameters, the sums of the squares of the differences between the simulated and experimentally derived durations and peak velocities were calculated. These were normalised by the variances of the experimentally derived values for duration and peak velocity, respectively, to give equal weight to the duration and peak velocity data. The final values selected for $\kappa$ and $\lambda$ were those that minimised the sum of the duration and peak velocity scores. Given these values of $\kappa$ and $\lambda$, the values of $\mu$ required to produce saccades of 5$^{\circ }$–25$^{\circ }$ in 5$^{\circ }$ steps were found by successively adjusting the values of $\mu$ that were originally used to sweep over $\kappa$ and $\lambda$. The procedure used to estimate $\kappa$ and $\lambda$ was then repeated to check that they were the same with the new set of values for $\mu$.

**Table 2:** Parameter values used to fit the data with the slow–fast saccadic Model 1, described by Eq. (4)

Species	$\kappa$	$\lambda$	$\mu$
Human	500	0.018	$0.218+0.223 \sqrt{A}$
Rhesus	620	0.013	$0.230+0.232\sqrt{A}$
Cat	140	0.014	$0.150 -0.050 A +0.619\sqrt{A}$
Rabbit	270	0.030	$0.228 +0.231\sqrt{A}$
Mouse	240	0.001	$1.511 -0.035 A +0.376\sqrt{A}$

$\kappa$ is the output scale factor used in the equation for the oculomotor integrator. $\lambda$ is the time constant introduced to account for interspecies differences in saccade speed and $\mu$ is the factor used to scale the output of the accumulator unit to obtain a saccade of specific size. *A* denotes saccade amplitude in degrees

The parameter values giving the best fit to the experimental data for Model 1 are given in Table 2, and the main sequence fits are shown in Fig. 4a. The error between the modelled values of duration and peak velocity at 5$^{\circ }$ intervals and the experimental data was expressed as a percentage of the value of the experimental data, and the mean of these percentages is given in Table 4 (left column).

**Table 3:** Parameter values used to fit the data with the slow–fast saccadic Model 2 described by Eq. (5)

Species	$\kappa$	$\lambda$	$\mu$	$\theta$
Human	500	0.018	$0.218+0.223 \sqrt{A}$	1.0
Rhesus	840	0.011	$0.170+0.064\sqrt{A}$	2.0
Cat	750	0.1	$0.495+0.374\sqrt{A}$	0.4
Rabbit	300	0.030	$0.192 +0.123\sqrt{A}$	1.4
Mouse	1200	0.003	$0.094 +0.023\sqrt{A}$	5.0

$\theta$ is a parameter used to alter the eigenvalues at the fixed point without altering its position. $\kappa$ is the output scale factor used in the equation for the oculomotor integrator. $\lambda$ is the time constant introduced to account for interspecies differences in saccade speed, and $\mu$ is the factor used to scale the output of the accumulator unit to obtain a saccade of specific size. *A* denotes saccade amplitude in degrees

It can be seen that the simulated results replicate the range of behaviours found in the human, rhesus monkey and rabbit, but the model was unable to accurately reproduce the eye movements of the mouse and cat, giving poorer approximations to the required relations between peak velocity and duration, as indicated by the significantly higher fitting error for these species. The fit for all species was best with 15$^{\circ }$ saccades, and the profiles of these saccades are plotted in Fig. 4b. This figure shows that whilst the human, rhesus monkey and rabbit saccades have the expected symmetrical velocity profiles, those of the mouse and cat do not.

The parameter values yielding the best fits with Model 2 were found by repeating the procedure implemented for Model 1, using the values given in Table 2 for the initial values of the search, with values of $\theta$ separated by intervals of 0.2. The resulting best-fit parameter values are listed in Table 3, and the main sequence fits are shown in Fig. 5a. The error between the simulated and experimental values of duration and peak velocity is reported in Table 4 (right column). For all species except the cat, the mean percentage error is less than 10%, demonstrating that Model 2 can replicate the range of saccadic behaviours found experimentally, with higher overall accuracy than Model 1. The fit for all species was again best with 15$^{\circ }$ saccades, and the profiles of the latter are plotted in Fig. 5b.

### Quantitative predictions of saccadic control signals

The geometric approach to the saccadic mechanism also leads to novel techniques for analysing measurements made on the system. Given a state-space reconstruction of the behaviour of the system, geometric analysis of the trajectories associated with individual saccades can then be used to identify equilibria and to approximate the local behaviour around each equilibrium with a set of first-order linear differential equations derived from the eigenvalues and eigenvectors of the linearised vector field. This approach has previously been shown to be applicable to eye movement recordings (Abadi et al. 1997; Akman et al. 2006; Theodorou and Clement 2007; Akman et al. 2012).

**Table 4:** Mean errors of Models 1 and 2 expressed as a percentage of the experimentally determined durations and peak velocities

Species	Model 1		Model 2	
	Duration (%)	Velocity (%)	Duration (%)	Velocity (%)
Human	5.7	5.3	5.7	5.3
Rhesus	9.9	9.9	5.5	4.8
Cat	16.9	16.4	12.5	12.3
Rabbit	9.0	4.2	8.0	5.0
Mouse	24.4	27.4	6.1	6.2

**Fig. 6:**
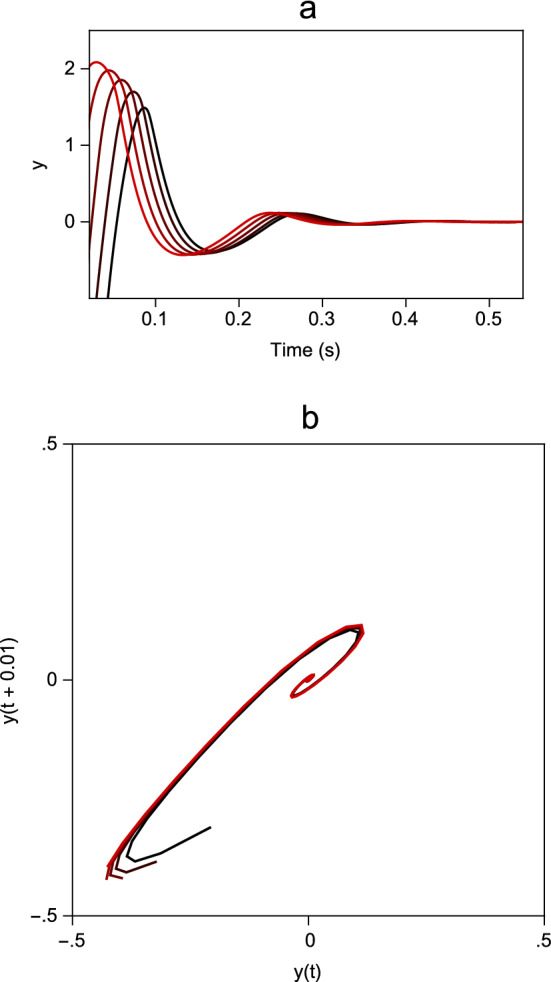
Illustration of how measurements of the velocity command can be used to find the eigenvalues of the reconstructed linear dynamics of Model 2, with the parameters for a human (Table 3), close to the equilibrium position. **a** Plot of the *y* variable timeseries used to generate 5 saccades ranging from 5$^{\circ }$ to 25$^{\circ }$ in amplitude. **b** Corresponding reconstructed system trajectories in a two-dimensional delay space (colour figure online)

To apply this approach, for example, to the behaviour of Model 2, the first step is to compute the time course of the *y* variable for saccades of various sizes, as illustrated in Fig. 6a. Next, a two-dimensional delay space representation of the system trajectory is generated by taking successive pairs of *y* values separated by intervals of 10 ms, as illustrated in Fig. 6b. Given this state-space representation, one can use the collection of delay space points within a small radius of the equilibrium to construct a local linear model of the system. Close to the resting equilibrium position, the system spirals in towards the equilibrium so the behaviour is characterised by a pair of complex conjugate eigenvalues. The eigenvalue corresponding to the fast movement transverse to the slow manifold cannot be recovered by this technique because the points have already contracted onto the manifold. For comparison, the eigenvalues calculated directly from Eq. (5) and the eigenvalues calculated from the delay space reconstruction are listed in Table 5. Although the simplest delay space reconstruction (i.e. using an embedding dimension of two) has been used to illustrate the technique, it can be seen that the exact and reconstructed eigenvalues are very similar, with the exception of the real part of the eigenvalue for the cat. This being the case, one would expect to be able to recover the eigenvalues of the local linear behaviour from recordings of medium-lead burst neuron firing rates.

**Table 5:** Real and imaginary parts of the complex conjugate pair of eigenvalues $\left\{ \xi ,\bar{\xi }\right\}$ that describe how the state of Model 2, with the parameters for a human, spirals towards its equilibrium

Species	Calculated		Reconstructed	
	Re ($\xi$)	Im ($\xi$)	Re ($\xi$)	Im ($\xi$)
Human	$-$ 13.8	36.7	$-$ 14.1	36.5
Rhesus	$-$ 22.6	39.4	$-$ 21.6	40.4
Cat	$-$ 2.4	10.9	$-$ 4.5	13.3
Rabbit	$-$ 8.3	18.1	$-$ 8.2	18.8
Mouse	$-$ 83.3	64.6	$-$ 75.5	68.2

One set of values was calculated directly from Eq. (5) and the other set was calculated from the trajectories in delay space reconstructed from the velocity command, *y*(*t*)

### Qualitative simulation of omnipause cell behaviour

As well as generating saccades, the brainstem oculomotor circuitry has a number of features that any model of its behaviour needs to be compatible with. In particular, electrical stimulation and chemical lesions of the omnipause neurons have characteristic effects on the behaviour of the oculomotor system and so provide an important challenge for any model. Simulating the effects of stimulation or lesions requires further simplifying assumptions which may not be valid. Our goal in this section is therefore simply to show how changes in the behaviour of Model 2 [Eq. (5)] can occur that mimic the behavioural changes found experimentally. Another characteristic property of pause cells is that the firing rate of approximately half of them is negatively correlated with pursuit eye velocity (Missal and Keller 2002) and in this section we also place this behaviour within the context of slow–fast dynamics.

Because we have used the output of the *z* variable to reset the accumulator, alterations to the behaviour of this variable to simulate experimental manipulations of the pause neurons sometimes results in failure to reset the accumulator. Hence, to ensure that only one saccade occurred, we subtracted a constant of $\nicefrac {1}{2}$ from the *z* variable in the accumulator equation. Eq. (5) thus becomes

6 $$\begin{aligned} \begin{aligned} \lambda \dot{a}&= H(a)\left( z-\frac{1}{2}\right) , \\ \lambda \dot{x}&= -y-1, \\ \lambda \dot{y}&= -y-z -\mu a,\\ \lambda \epsilon \dot{z}&= -( \theta (z^{3}+y z)+x),\\ \dot{n}&= -\frac{n}{T_{n}}+\kappa y^{+}, \end{aligned} \end{aligned}$$

and the version of Model 2 used in this section consists of Eq. (6), which we refer to collectively as Model 2*.

Electrical stimulation of the omnipause neurons was modelled by adding a smoothed pulse of excitation to the right-hand side of the *z* equation in (6) that lasted from 30 to 60 ms after the onset of a 25$^{\circ }$ saccade. The shape of the pulse was designed to model an abrupt change in stimulation but with smooth onset and offset to ensure convergence of the numerical solution of the equations. A relatively simple function, *g*(*t*), which satisfies these requirements is given by:

7 $$\begin{aligned} \begin{aligned} g(t)&= G\left( 1- \displaystyle {\frac{(t-\tau _l)^{m}}{\tau _w^{m}+(t-\tau _l)^{m}}}\right) . \end{aligned} \end{aligned}$$

**Fig. 7:**
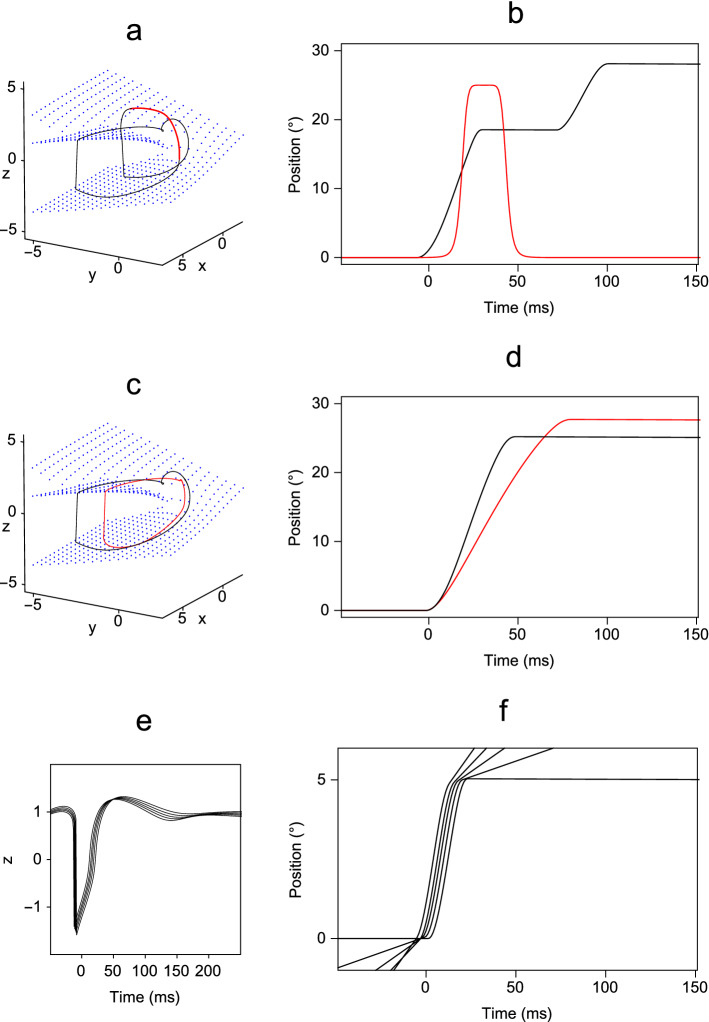
Simulations of experimental manipulations of the pause cells generated using Model 2* [Eq. (6)] with the parameter values for a rhesus monkey (Table 3). Top row: Effect of electrically stimulating the pause cells during a 25$^{\circ }$ saccade by adding a pulse function specified by Eq. (7) to the *z* variable in (6), with the following parameter values: $G=30$, $\tau _l=0.13$, $\tau _w=0.0125$, $m=8$. **a** Phase space trajectory of the *x*, *y* and *z* variables—the red line indicates the portion of the trajectory for which the pulse is on. **b** Plot of eye position *n*(*t*) against time. The time course of the pulse is overplotted in red (arbitrary units). Middle row: Effect on a 25$^{\circ }$ saccade of chemically lesioning the pause cells by halving their contribution to the equation specifying the *y* variable in Eq. (6). **c** Phase space trajectory of the *x*, *y* and *z* variables—the black line illustrates a normal saccade and the red line illustrates the slowed saccade. **d** Plot of eye position against time for a normal saccade (black line) and the slowed saccade (red line). Bottom row: Reduction of pause cell resting level required for catch-up saccades of amplitude 5$^{\circ }$ to be on target during smooth pursuit, with pursuit velocities of 0, 20, 40, 60 and 80 $^{\circ }$ s$^{-1}$. **e** Plots of the *z* variable against time generated with the offsets to the *x* equation in (6) (1,0.973, 0.95, 0.93, 0.91) required to reduce the size of the resulting saccade command (5$^{\circ }$, 4.47$^{\circ }$, 4.08$^{\circ }$, 3.76$^{\circ }$ and 3.37$^{\circ }$, respectively). The value of $\mu$ used was 0.388 in all cases. **f** Corresponding plots of eye position *n*(*t*) against time. We have selected the vertical range to match the size of the saccades so that it is clear that the offset results in correctly calibrated saccades (colour figure online)

To assess the effect of adding such a pulse, saccades were simulated with the pulse amplitude, *G*, varying from 10$^{\circ }$ to 50$^{\circ }$, the pulse onset time $\tau _l$ varying from 120 to 140 ms, the width parameter $\tau _w$ from 10 to 15 ms and the steepness parameter *m* from 6 to 10. In all cases, the pulse caused a mid-flight interruption to the saccade. An example simulation is illustrated in Fig. 7b. The effect of the pulse is to force the state of the system to move rapidly from the negative to the positive part of the slow manifold, whereupon it moves relatively slowly along the manifold towards the equilibrium point specified by the pulse. As the vector fields on the two portions of the manifold are similar, as soon as the pulse ends, the state changes along the upper portion of the manifold are mirrored in the lower portion of the manifold, and the saccade restarts from approximately the same state that it was in at the time of the pulse onset. This dynamical behaviour is illustrated in Fig. 7a. The experimental findings on the effect of electrical stimulation are that the resulting saccades are hypermetropic (Keller et al. 1996). In our simulation they varied from being hypometropic (saccade amplitude less than required) to hypermetropic (saccade amplitude more than required), depending on the pulse parameters. This is because our simplified model takes no account of the physical interaction of the pulse with the neurons and is intended only to show that mid-flight pausing of saccades is an expected consequence of temporarily increasing the *z* variable in Model 2*.

Chemical lesioning of the pause cells results in slowing of saccades, without loss of accuracy (Kaneko 1966). We modelled the damage to the pause units by reducing the *z* variable to a half of its original value in the specification of the *y* equation in (6). An example of the resulting slowed saccade is shown in Fig. 7d. Unlike its experimentally observed counterparts, the simulated saccade is no longer accurate. This is to be expected, however, as the experimentally observed saccades continue to be accurate because the longer durations give enough time for visual feedback to be used to correct the amplitude discrepancy (Zee et al. 1976), and this compensation mechanism is not included in our model. The effect of the change in the equations is to slow the spiral of the portion of the trajectory lying on the slow manifold, as shown in Fig. 7c.

If a target is moving slowly and smoothly the pursuit eye movement system is able to follow it closely, but with increased target velocity the eye position frequently lags behind the target position. In such cases small saccades, referred to as catch-up saccades, are made to bring the eye position into alignment with the target position. Such a catch-up saccade must have its amplitude reduced by the distance travelled by the pursuit component of the eye movement during the saccade, and measurements of the main sequence during pursuit confirm that this reduction occurs (DeBrouwer et al. 2002). The amplitude of a saccade can be reduced by changing the constant in the equation for the *x* variable in (6), which has a nominal value of 1. If the constant is reduced, then the equilibrium point is shifted so that the required amplitude of the saccade signalled by the *x* variable is decreased, but so is the value of the *z* variable corresponding to the pause cell firing. Examples of this compensation process for pursuit at constant velocity are illustrated in Fig. 7f. The majority of catch-up saccades are less than 5$^{\circ }$, so this was chosen for the amplitude of the example saccade. The example pursuit velocities ranged from 0 to 80$^{\circ }$ s$^{-1}$ in 20 $^{\circ }$ s$^{-1}$ steps to match the velocities used in the study of Missal and Keller (2002). Pursuit was simulated in each case by adding the velocity value to the right-hand side of the equation for the oculomotor integrator. The corresponding reduction in the resting levels of pause cell activity which results from the shift of the equilibrium point is illustrated in Fig. 7e. Although the reduction in pause cell activity was highly correlated with the pursuit velocity, as is found experimentally, the reduction was much less than that found experimentally. For targets moving at 40$^{\circ }$ s$^{-1}$, it was found that the pause cell firing rate was reduced by 34% on average (Missal and Keller 2002), whereas in our simulations the reduction was only 5%. This finding implies that the initial guess at the form of the slow manifold is not exactly correct and needs to be experimentally determined.

We conclude that if it is assumed that pause cells are components of a circuit embodying Zeeman’s model for an action, then the properties of stimulation causing mid-flight halting, lesioning causing slowing and reduction of firing during smooth pursuit are all expected features of the behaviour of the circuit.

## Discussion and future directions

### Conceptualisation of the saccadic mechanism

Saccadic eye movements are remarkable for their consistency throughout life and provide a relatively simple and accessible example of discrete behavioural events. We have used them to test a general theory of such events that was developed by Zeeman (1977) and began by testing whether appropriate extensions of Zeeman’s basic model could replicate the experimentally determined main sequences of five animal species. Although Model 1, comprised of Eq. (4), yielded reasonable simulations of main sequence data in humans, rhesus monkeys and rabbits, it gave poorer approximations to the main sequences of the cat and mouse. The greater flexibility of Model 2, comprised of Eq. (5), resulted in good approximations to the experimental data, except in the case of the cat. This may in part be due to the greater variability of the saccadic trajectories of the cat (Evinger and Fuchs 1978), but no clear indication of the presence of a different mechanism emerged from this modelling work.

A local feedback mechanism, in which the firing of the medium-lead burst neurons depends on the difference between the target displacement of the eye and an internally generated estimate of current eye displacement, has proved highly successful in explaining saccadic eye movement behaviour (Scudder et al. 2002; Sparks 2002). To explain the presence of the omnipause neurons within this framework, it is assumed that they ensure the burst cells are quiescent when saccades are not being made. This assumption results in a requirement for a trigger mechanism that provides a pulse of activity that switches off the pause cells at the start of a saccade and a latch mechanism that ensures the pause units are kept silent throughout the duration of the saccade.

By contrast, within the framework for describing an action proposed by Zeeman (1977), the task of the brainstem circuitry controlling saccadic eye movements is to specify a trajectory corresponding to an action, and the build-up, burst and pause neurons are components of the simplest generic circuit which can perform this computation. The idea that the circuitry specifies the movement trajectory, and not just the endpoint, has come to the fore with the work of Goossens and Van Opstal (2006), who found that collicular burst cells produce the same number of spikes, even when the saccade is perturbed by a blink, implying that the firing of the burst cells is directly related to the progress of the saccadic movement along its trajectory. However, although the slow–fast framework does not explicitly require trigger and latch mechanisms, in practice the distinction is not so clear-cut. This is because the proposed trigger mechanism involves inhibition of the pause cells by the long-lead burst neurons and the proposed latch mechanism consists of mutual inhibition between the burst neurons and omnipause neurons (Van Gisbergen et al. 1981), and both of these relationships (inhibition of the *z* variable by the *x* variable and mutual inhibition of the *y* and *z* variables) are also part of the slow–fast model.

Our results on simulation of the main sequence do not depend on inclusion of the accumulator as we could have just followed Zeeman’s approach and specified a threshold displacement of the state of the system for each size of saccade. But we also wanted to explore how the mechanism of action generation links in with the decision making process of which action to make. Within the dynamical systems framework, the effect of the accumulator is to move the state of the system up towards the fold and the size of the saccade depends only on the position at which the trajectory leaves the fold. This behaviour is similar to that recently found in the motor cortex during voluntary movement (Churchland et al. 2012) in which the preparatory neural activity produces a specific oscillatory activity, even with discrete actions. If this analogy holds, then the oscillatory activity corresponds to the spiral trajectory necessary to move the state round the fold to the equilibrium position in the Zeeman model.

### Neurophysiological correlates

**Fig. 8:**
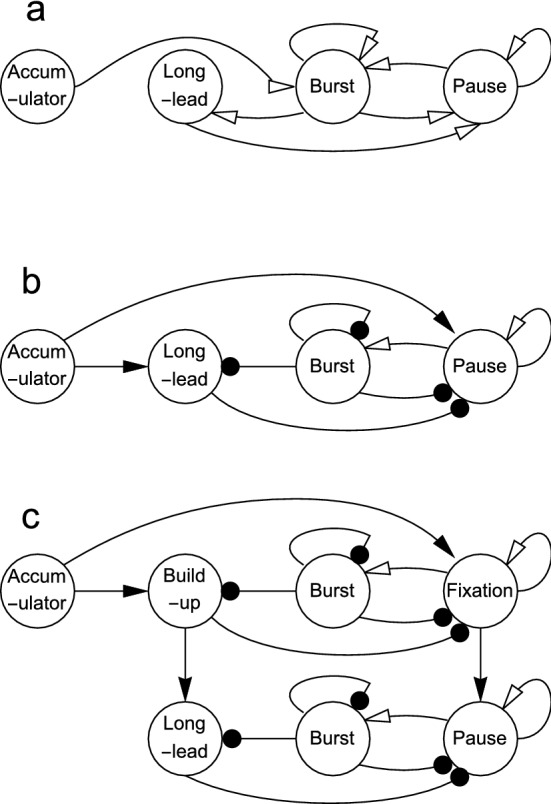
Possible neural circuits for implementation of slow–fast control of saccades. **a** Connections corresponding to Eq. (3). The unfilled arrows signify that the signal can be either positive or negative. **b** Connections corresponding to Eq. (8). The filled arrows signify excitatory connections and the filled circles signify inhibitory connections. **c** Possible extension of the circuit in **b** to include the superior colliculus

Our modelling of the brainstem saccadic circuitry began with the hypothesis that the variables *x*, *y* and *z* in Eq. (3), upon which our Models 1, 2 and 2* were built, play roles corresponding to the long-lead burst neurons, medium-lead burst neurons and pause neurons, respectively. The most direct interpretation of the model equations is that each term in Eq. (3) corresponds to a connection between the three types of neurons. This interpretation leads to the circuit diagram illustrated in Fig. 8a. The obvious drawback of this interpretation is that the behaviour of the model then requires some connections to be both excitatory and inhibitory which is not physiologically realistic. So, we further assumed that the sign associated with each term specifies whether the corresponding connection is inhibitory or excitatory and assessed when this is the case and what alterations can be made to the connections to implement a neurophysiologically plausible circuit.

One possible exception to the requirement that a connection must be either excitatory or inhibitory is that of the *z* variable to the *y* variable, which corresponds to the input from the pause cells to the medium-lead burst cells. According to Eq. (3), when the *z* variable is negative it increases the *y* variable, so the pause cells should also be able to increase the firing rate of the medium-lead burst cells. A possible neurophysiological mechanism for this change has been put forward by Optican (2008), who pointed out that omnipause neurons produce the transmitter glycine, which could act as an inhibitor on the burst cells when they are quiescent and could amplify their response when they are firing.

The *x* variable both increases or decreases depending on the sign of the *y* variable according to Eq. (3), and there is no evidence for these excitatory and inhibitory connections from the medium-lead to the long-lead burst neurons. The requirement for both inhibitory and excitatory connections can be eliminated by having separate neural pathways for the positive and negative values of the *y* variable.

One approach to achieving this is by assuming that the accumulator output feeds directly into the long-lead burst neurons, rather than indirectly via the medium-lead burst neurons. As the negative values of the *y* variable also ensure that the *z* variable remains positive during the build-up to the saccade initiation, the accumulator output must also be supplied to the pause neurons. In effect, the *y* variable is replaced by the difference $y -\mu a$ between the positive *y* and accumulator *a* variables on the right-hand side of Eq. (3). Even with these changes, all the *y* variable effects are a combination of excitatory and inhibitory effects because the system settles back to an equilibrium point where the value of *y* is $-1$. This aspect of the behaviour of the *y* variable can be eliminated by translating the slow manifold along the *y* axis so that the equilibrium point becomes (0, 0, 1). This translation is equivalent to substituting $y-1$ for *y* throughout Eq. (3). With these changes, the equations for Model 2 become:

8 $$\begin{aligned} \lambda \dot{a}= & {} H(a)\left( z-\frac{1}{2}\right) , \nonumber \\ \lambda \dot{x}= & {} -(y-\mu a), \nonumber \\ \lambda \dot{y}= & {} -(y -1)-z,\nonumber \\ \lambda \epsilon \dot{z}= & {} -(\theta (z^{3}+z(y-1-\mu a))+x),\nonumber \\ \dot{n}= & {} -\frac{n}{T_{n}}+\kappa (y-1)^{+}. \end{aligned}$$

**Fig. 9:**
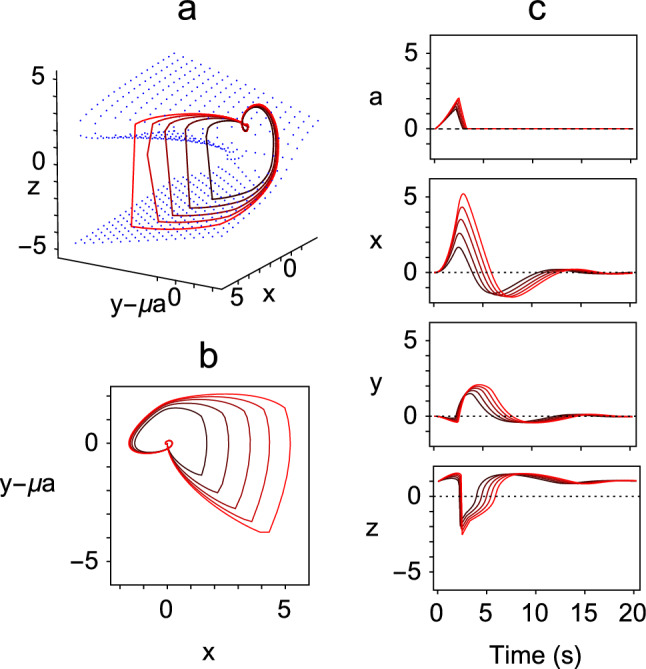
Example solutions of Eq. (8) obtained with $\lambda =1$ and $\mu =1.122,\,1.334,\,1.476,\,1.586,\,1.677$. **a** Phase space plot showing the trajectories of the *x*, $y-\mu a$ and *z* variables, in which the slow manifold is indicated by blue dots. By comparison with Fig. 2a, the difference $y-\mu a$ between the *y* variable and the accumulator variable is used to recreate the behaviour of the original *y* variable. **b** Projections of the trajectories onto the $(x,y-\mu a)$ plane. **c** Timeseries plots of *a*, *x*, *y* and *z*, showing the characteristic behaviour of each variable throughout the range of values of $\mu$ (colour figure online)

We note that because the offset added to the *y* variable is only exactly correct in the steady-state condition, the depth of inhibition of the accumulator again had to be increased by subtracting an offset of $\nicefrac {1}{2}$ from the *z* variable to ensure that the accumulator resets completely, as was done for Model 2*. The behaviour of this system is similar to that of Eq. (3), as illustrated in Fig. 9. The main sequence behaviour is also in correspondence, provided that the positive values of $y-1$, rather than *y*, are used as the signal to the oculomotor integrator. It can be seen that although both the *x* and *y* variables still show transient changes in sign, these occur after the action has taken place and are not critical to the slow–fast model (Fig. 9c). The corresponding circuit diagram is shown in Fig. 8b.

Aside from the connections from the accumulator, there is physiological evidence for the majority of the connections in Fig. 8b. Long-lead burst neurons (*x*) are found in the central mesencephalic reticular formation, which lies between the superior colliculus and the nucleus raphe interpositus, where the omnipause neurons are situated (Cromer and Waitzman 2006). Labelling studies have further revealed that there are inhibitory connections from the central mesencephalic reticular formation to the nucleus raphe interpositus (Wang et al. 2013). These connections correspond to the required inhibition of the *z* variable by the *x* variable in Eq. (3). Medium-lead burst cells (*y*) inhibit the pause cells (*z*) because the level of hyperpolarisation of the pause cells matches the firing pattern of burst cells during saccades (Yoshida et al. 1999; Van Horn et al. 2010). The pause cells inhibit the burst cells, because stimulation of the pause cells stops an ongoing saccade. However, there is no evidence at present for a connection corresponding to the input from the *y* variable to the *x* variable, and this is a key connection because the *x* variable performs the role of carrying a displacement signal that is reset by integrating the velocity signal carried by the *x* variable.

The assumptions of an accumulator unit, together with their abrupt resetting by pause cells, were made simply to ensure a gradual build up of the sensory input. To illustrate how a more comprehensive description of the sensory component of the saccadic mechanism might be developed, we present a diagram of a combined brainstem and superior colliculus circuit in Fig. 8c. The modification of the circuit to involve direct input to the *x* variable from the accumulator has implications for this combined circuit. Firstly, the required displacement in the brainstem can be set up independently of the *y* variable so the latter variable can be rescaled leading to faster or slower saccades with the same amplitude. Secondly, one role of the excitatory input to the pause cells in the brainstem may be to ensure that the state of the system remains on the slow manifold in the build-up to the saccade.

The strength of the linear terms in Eq. (8) could be determined by finding the predicted eigenvalues, as outlined in the Results section. A reconstruction of the entire trajectory of the brainstem burst generator could, in principle, be obtained by using simultaneous recordings from the long-lead burst neurons, medium-lead burst neurons and pause cells as coordinates for points in a three-dimensional state space. If simultaneous recordings of the three neuron types are not available, then an alternative approach would be to synchronise recordings from individual neurons by aligning them with the associated eye movement recordings. The reconstructed trajectories are predicted to be similar to those illustrated in Fig. 9, enabling testing of how well the form of the slow manifold of the slow–fast saccadic model (5) matches that of the real oculomotor system.

## Conclusions

In summary, we have shown that a slow–fast model of the saccadic system can account for the behaviour of the system, whilst being compatible with the brainstem physiology. We have also provided predictions of the stability of the resting position at the end of the saccade so that the validity of the model can be experimentally tested. The experimental testing is important because the model implies a new role for the pause cells that does not involve simply shutting down the burst neurons.
